# Draft Genome Sequence of the Yeast Blastobotrys aristata Strain UCD613, Isolated from Soil in Ireland

**DOI:** 10.1128/mra.00957-22

**Published:** 2022-10-12

**Authors:** Aida Don, Kevin P. Byrne, Fouz Alqaderi, Alexandra Mazhova, Ben O’Leary Chaney, Sarah Redmond, Stephen Allen, Jiaran Gao, Eoin Ó Cinnéide, Sean A. Bergin, Conor Hession, Kenneth H. Wolfe, Geraldine Butler

**Affiliations:** a School of Biomolecular and Biomedical Science, Conway Institute, University College Dublin, Belfield, Ireland; b School of Medicine, Conway Institute, University College Dublin, Belfield, Ireland; Vanderbilt University

## Abstract

Blastobotrys aristata is a member of the Trichomonascaceae family in the order Saccharomycetales. Here, we present the genome sequence of *B. aristata* UCD613, which was isolated from soil in Dublin, Ireland. This genome is 13.3 Mb and was assembled into 4 chromosome-size scaffolds of >2.2 Mb in size plus a mitochondrial genome scaffold.

## ANNOUNCEMENT

Blastobotrys aristata was first isolated from moldy plaster in the former Czechslovakia in 1976 (Marvanova 1976, also known as *B. aristatus*) (1). We identified isolate *B. aristata* UCD613 from soil collected from the campus of University College Dublin (GPS coordinates 53.3034961, −6.2131910). Soil material was passaged twice in in 9 mL liquid yeast extract-peptone-dextrose (YPD) containing chloramphenicol (30 μg/mL) and ampicillin (100 μg/mL) and cultured on YPD plates at 30°C.

The species was identified from single colonies by PCR amplification and Sanger sequencing of the internal transcribed spacer (ITS) (OP221981) and D1/D2 (OP221771) regions of its ribosomal DNA (rDNA) locus. The D1/D2 region was 100% identical to that of the type strain of *B. aristata* (2) (DQ442686.1). No other ITS sequence is available.

For short-read sequencing, total genomic DNA was extracted from a YPD culture using phenol-chloroform-isoamyl alcohol and dissolved in 150 μL water (3). Libraries were generated and sequenced by BGI Tech Solutions (Hong Kong). One microgram of DNA was fragmented using Covaris, size selected (200 to 400 bp) using magnetic beads, end repaired, and 3′ adenylated, and primers were ligated. Fragments were amplified by PCR and heat denatured and circularized using the splint oligonucleotide sequence. The library was amplified with ϕ29 DNA polymerase to make DNA nanoballs (DNBs). The DNBs were loaded on a patterned nanoarray, and 150 bases were sequenced from each end using combinatorial probe-anchor synthesis (cPAS) on a DNBSeq-G400, yielding ~6.1 million read pairs. Default parameters were used unless noted. Adapters and low-quality reads were removed first using SOAPnuke (4) and subsequently using Skewer v.0.2.2 (5). For long-read sequencing, genomic DNA was prepared using a Genomic Tip 100G kit (Qiagen). Two libraries were generated using the SQK-RBK004 kit from Oxford Nanopore Technologies (ONT) and cleaned with AMPure XP magnetic beads. Libraries were sequenced on primed R9.4.1 flow cells using MinKNOW v.4.1.22 on a MinION device. From run 1, raw data were base called using Guppy v.4.2.2 +effbaf8 (using the fast model [dna_r9.4.1_450bps_fast.cfg]) (ONT) and demultiplexed using qcat v.1.1.0 (ONT) with default settings. For the second run, Guppy v.4.2.2 +effbaf8 was used both for base calling and demultiplexing. Both sets of reads were concatenated together for downstream processing. NanoFilt v.2.3.0 (6) was used to select reads (minimum quality, ≥7; minimum length, ≥1,000 bp) which retained 107,000 reads with an *N*_50_ of 6,639 bp.

The genome was assembled from the long reads using Canu v.2.2 (7), followed by five rounds of error correction with the DNBseq short reads using NextPolish (8). Five contigs of <45 kb (corresponding to rDNA and parts of the mitochondrial genome) were removed, leaving 4 chromosome-size contigs of >2.2 Mb in size and a circular mitochondrial genome (48,582 bp, manually edited; accession no. OX291664.1). The total size of the genome is 13.3 Mb, the *N*_50_ value is 3.5 Mb, the *L*_50_ value is 2 contigs, and the G+C content is 48%. The largest contig is 4.2 Mb. Using BUSCO v.5.1.2, genome completeness was estimated at 94.8% (compared to the Ascomycota lineage data set).

### Data availability.

This whole-genome shotgun project has been deposited at DDBJ/ENA/GenBank (BioProject no. PRJEB55420). The version described in this paper is version 1. The raw reads were deposited at SRA (accession no. ERX9629577, ERX9629578, and ERX9629579). The ITS sequence is at OP221981 and the D1/D2 region sequence at OP221771.
